# Down-regulation of AMPK signaling pathway rescues hearing loss in TFB1 transgenic mice and delays age-related hearing loss

**DOI:** 10.18632/aging.102977

**Published:** 2020-04-02

**Authors:** Jingjing Zhao, Gen Li, Xuan Zhao, Xin Lin, Yunge Gao, Nuno Raimundo, Geng-Lin Li, Wei Shang, Hao Wu, Lei Song

**Affiliations:** 1 Department of Otolaryngology, Head and Neck Surgery, Shanghai Ninth People’s Hospital, Shanghai Jiao Tong University School of Medicine, Shanghai, China; 2 Ear Institute, Shanghai Jiao Tong University School of Medicine, Shanghai, China; 3 Shanghai Key Laboratory of Translational Medicine on Ear and Nose Diseases, Shanghai, China; 4 Navy Clinical Medical School, Anhui Medical University, Hefei, China; 5 Department of Otorhinolaryngology, Head and Neck Surgery, Zhongshan Hospital, Fudan University, Shanghai, China; 6 Institute of Cellular Biochemistry, University Medical Center Göttingen, Göttingen, Germany; 7 Department of Otorhinolaryngology, Eye and ENT Hospital, Fudan University, Shanghai, China; 8 In Vitro Fertility (IVF) Center Department of Obstetrics and Gynecology, the Sixth Medical Center of PLA General Hospital, Beijing, China

**Keywords:** mitochondrial deafness, ROS, AMPK, apoptosis, NIHL

## Abstract

AMP-activated protein kinase (AMPK) integrates the regulation of cell growth and metabolism. AMPK activation occurs in response to cellular energy decline and mitochondrial dysfunction triggered by reactive oxygen species (ROS). In aged Tg-mtTFB1 mice, a mitochondrial deafness mouse model, hearing loss is accompanied with cochlear pathology including reduced endocochlear potential (EP) and loss of spiral ganglion neurons (SGN), inner hair cell (IHC) synapses and outer hair cells (OHC). Accumulated ROS and increased apoptosis signaling were also detected in cochlear tissues, accompanied by activation of AMPK. To further explore the role of AMPK signaling in the auditory phenotype, we used genetically knocked out AMPKα1 as a rescue to Tg-mtTFB1 mice and observed: improved ABR wave I, EP and IHC function, normal SGNs, IHC synapses morphology and OHC survivals, with decreased ROS, reduced pro-apoptotic signaling (Bax) and increased anti-apoptotic signaling (Bcl-2) in the cochlear tissues, indicating that reduced AMPK attenuated apoptosis via ROS-AMPK-Bcl2 pathway in the cochlea. To conclude, AMPK hyperactivation causes accelerated presbycusis in Tg-mtTFB1 mice by redox imbalance and dysregulation of the apoptosis pathway. The effects of AMPK downregulation on pro-survival function and reduction of oxidative stress indicate AMPK serves as a target to rescue or relieve mitochondrial hearing loss.

## INTRODUCTION

A highly evolutionarily conserved serine/threonine kinase — adenosine 5’-monophosphate activated protein kinase (AMPK), is a core regulator of cellular and organismal metabolism [1], which coordinates catabolic with anabolic pathways to maintain the levels of intracellular ATP [2] and is recognized as a cellular energy sensor [3]. AMPK has been proved as a heterotrimeric complex composed of a catalytic α subunit and two non-catalytic regulatory subunits, β and γ [4]. The kinase domain of the α subunit is regulated via upstream kinases promoting phosphorylation of threonine residue 172 (Thr172) site in the activation loop [5], which is indispensable for AMPK activation. Dysregulation of AMPK is associated with various body systems and human diseases, including cardiovascular diseases, type2 diabetes and neurodegenerative disorders [6] — Alzheimer’s disease, Parkinson’s disease, Huntington’s disease, inflammatory disorders, viral infection and cancer [7, 8]. AMPK can be activated by various cellular stress, including hypoxia, glucose deprivation, and reactive oxygen species (ROS) accumulation [9, 10], which are often mainly derived from mitochondria. Excessive mitochondrial ROS production results in damage of cellular lipids, proteins, and mitochondrial DNA, promoting cytotoxicity and induction of cellular senescence [11].

In a previously reported animal model Tg-mtTFB1 [12] (Tg-B1) that recapitulates mtDNA mutation A1555G, progressive hearing loss [13] occurred due to apoptosis in the cells of organ of Corti (OC) [14]. Superfluous ROS were produced in the cochlea results in dysfunction of SGNs and SV [12], coupled with enhanced up-regulation of AMPK signaling in tissue-specific fashion via oxidative stress and subunit AMPKα1 [15]. Meanwhile, hyper-methylation of mitochondrial ribosomes proved to be a critical molecular defect driving the apoptotic phenotype and deafness in Tg-B1 mice induced by a pro-apoptotic, ROS-AMPK-E2F1 pathway [14], which manifested as increased E2F1 and apoptosis in the Stria Vascularis (SV) and SGNs of the inner ear, and progressive E2F1-dependent hearing loss. To further address the involvement of AMPK signaling in the pathogenic mechanism of hearing loss in Tg-B1 mice, we generated a genetic rescue of Tg-B1 by reducing AMPKα1 (AMPK^+/−^/Tg-B1). The AMPK het-KO mice exhibit normal ABR threshold compared to their wild type (WT) littermate controls, and the rescued mice had improved auditory phenotype compared to their littermate Tg-B1 mice at 10 to 12 months of age, implying that reducing AMPK signaling could rescue or delay age-related hearing loss in the transgenic Tg-B1 mouse model of mitochondrial deafness [12]. We here explored tissue-specific pathology and molecular events occurred in apoptosis signaling that rescued hearing phenotype.

## RESULTS

### Aging AMPK KO mice maintain better hearing sensitivity compared to Tg-B1 mice and WT littermate controls

**Figure 1:**
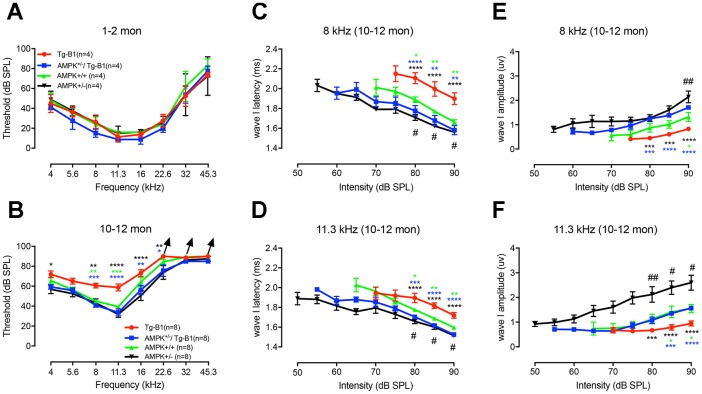
**General ABR findings reveal the protective effect of AMPK KO.** Auditory thresholds were evaluated by ABRs at age of 1-2 months (**A**) and 10-12 months (**B**) for four genotype groups. There is no significant difference in ABR thresholds for all four genotypes at 1-2 months (F_(3,12)_=2.972, p=0.0744, two-way ANOVA followed by Bonferroni post-test), while at 10-12 months of age, around 20dB threshold elevation was observed in Tg-B1 mice (red) at 8 kHz (Tg-B1 *vs.* WT, F_(1,14)_=28.974, p<0.001), 11.3 kHz (Tg-B1 *vs.* WT, F_(1,14)_=21.912, p<0.001, one-way ANOVA followed by Bonferroni post-test) compared to age-matched WT controls (green), and a moderate increase of thresholds at low frequencies. Age-matched AMPK^+/−^/Tg-B1 mice (blue) showed significantly lower ABR thresholds compared to Tg-B1 mice at 10-12 month, for 8 kHz (Tg-B1 *vs.* AMPK^+/−^/Tg-B1, F_(1,14)_ =50.479, p<0.001), 11.3 kHz (F_(1,14)_=25.455, p<0.001) and 16 kHz (F_(1,14)_=8.463, p=0.011, one-way ANOVA followed by Bonferroni post-test) and showed similar ABR thresholds to wild type controls (AMPK^+/−^/Tg-B1 *vs.* WT, F_(5,84)_=0.3781, p=0.8625, two-way ANOVA followed by Bonferroni post-test). However, there were no significant differences in ABR thresholds among AMPK^+/−^/Tg-B1 (blue), wild type controls (green) and AMPK^+/−^ (black) groups (F_(10,126)_=0.392, p=0.9482, two-way ANOVA followed by Bonferroni post-test). Arrowhead points excluded mice that showed no response at 90 dB SPL, the upper limit of the ABR recording. Number of mice with “no response” at 90 dB SPL: Tg-B1 mice at 22.6 kHz, n=2, 32 kHz, n=2, 45.3 kHz, n=3; AMPK^+/−^/Tg-B1 mice at 22.6 kHz, n=0, 32 kHz, n=1, 45.3 kHz, n=3; WT mice at 22.6 kHz, n=0, 32 kHz, n=1, 45.3 kHz, n=4 and AMPK^+/−^ mice at 22.6 kHz, n=0, 32 kHz, n=0, 45.3 kHz, n=3. (**C**–**F**) Amplitudes and latencies of ABR wave I in different genotype groups aged 10-12 months from 50-90 dB SPL (8 and 11.3 kHz) were computed from sorted ABR wave traces. In contrast to the AMPK^+/−^/Tg-B1 and wild type mice, latencies of ABR wave I are remarkably prolonged in Tg-B1 mice at 8 kHz (Tg-B1 *vs.* WT, F_(1,14)_=11.7, p=0.0041; Tg-B1 *vs.* AMPK^+/−^/Tg-B1, F_(1,14)_=15.71, p=0.0014; AMPK^+/−^ *vs.* WT, F_(1,14)_=19.84, p=0.0005, Figure 1C) and 11.3 kHz (Tg-B1 *vs.* WT, F_(1,14)_=14.91, p=0.0017; Tg-B1 *vs.* AMPK^+/−^/Tg-B1, F_(1,14)_=40.26, p<0.0001; AMPK^+/−^ *vs.* WT, F_(1,14)_=8.752, p=0.0104, two-way ANOVA followed by Bonferroni post-test, Figure 1D). Besides, significantly decreased amplitude of peak I was noticed in Tg-B1 mice at 8 kHz (F_(1,14)_=6.091, p=0.0271, Figure 1E) and 11.3 kHz (F_(1,14)_=7.792, p=0.0144, two-way ANOVA followed by Bonferroni post-test, Figure 1F) as compared to wild type controls. Significant increases of ABR wave I amplitude in AMPK^+/−^/Tg-B1 mice at both 8 kHz (F_(1,14)_=63.76, p<0.0001) and 11.3 kHz (F_(1,14)_=27.82, p=0.0001, two-way ANOVA followed by Bonferroni post-test) were also observed as compared to Tg-B1 mice. Briefly, AMPK^+/−^/Tg-B1 mice exhibited significantly increased wave I amplitudes (**E**, **F**) and shorter wave I latencies (**C**, **D**) as compared to those in Tg-B1 mice. Furthermore, AMPK^+/−^ mice (black) showed increased wave I amplitudes as compared to wild type mice (green) at both 8 kHz (F_(1,14)_=7.653, p=0.0151) and 11.3 kHz (F_(1,14)_=8.656, p=0.0107, two-way ANOVA followed by Bonferroni post-test), as marked with a pound sign (#). The bar graph represents the mean threshold/wave I amplitude or latency ± SEM (n=8). Asterisks symbolized statistically significant differences at the indicated frequencies and sound intensities.

Based on the previously characterized phenotype, Tg-B1 mice exhibited late-onset progressive hearing loss [14] and by downregulation of AMPK signaling, auditory phenotypes are rescued [12]. We followed the hearing function of the littermates of the four genotype mice listed above. There was no significant difference in ABR thresholds at the age of 1-2 months in the four young groups (Figure 1A), confirming the progressive nature of hearing loss. At 10-12 months of age, compared to wild type mice, ~20dB threshold elevations at 8 kHz, 11.3 kHz, and 16 kHz were observed in Tg-B1 mice (Figure 1B), with a mild increase of threshold at low frequencies (4 and 5.6 kHz, but non-significant). To determine the source of auditory dysfunction, detailed analysis of the ABR waves were conducted among the four genotype of mice aged 10-12 months (Figure 1C–1F). The latencies of ABR wave I reflect the elapsed time of synaptic transmission and nerve conduction, while the amplitudes of the wave I reflect the synchronized firing capability of the auditory nerve fibers [16]. As expected and in agreement with prior findings [12], in Tg-B1 mice, latencies of ABR wave I are remarkably prolonged and amplitudes of ABR wave I are significantly reduced at 8 kHz (Figure 1C and 1E) and 11.3 kHz (Figure 1D and 1F). AMPK^+/−^/Tg-B1 mice exhibited recovered wave I amplitudes (Figure 1E and 1F) and latencies (Figure 1C and 1D). The decreased amplitude of peak I in Tg-B1 mice at 8 kHz (Figure 1E) and 11.3 kHz (Figure 1F) are in good agreement with the reduced synaptic ribbons and SGN counts (Figure 3A and 3C). The decrease in wave I amplitude of Tg-B1 mice was rescued, as shown in AMPK^+/−^/Tg-B1 mice, which exhibited similar amplitudes to wild type controls at 11.3 kHz (Figure 1F) and even higher levels of average amplitudes than wild type controls at 8 kHz (Figure 1E). Furthermore, the auditory function of AMPKα1 het-KO mice is superior to wild type counterparts: with shorter latencies and more robust amplitudes of ABR wave I (Figure 1C–1F), suggest that the genetic knockout of AMPKα1 delays age-related hearing loss in C57BL/6J mice and has a protective effect on hearing in general.

### AMPK downregulation protects against sensory outer hair cells loss

**Figure 2:**
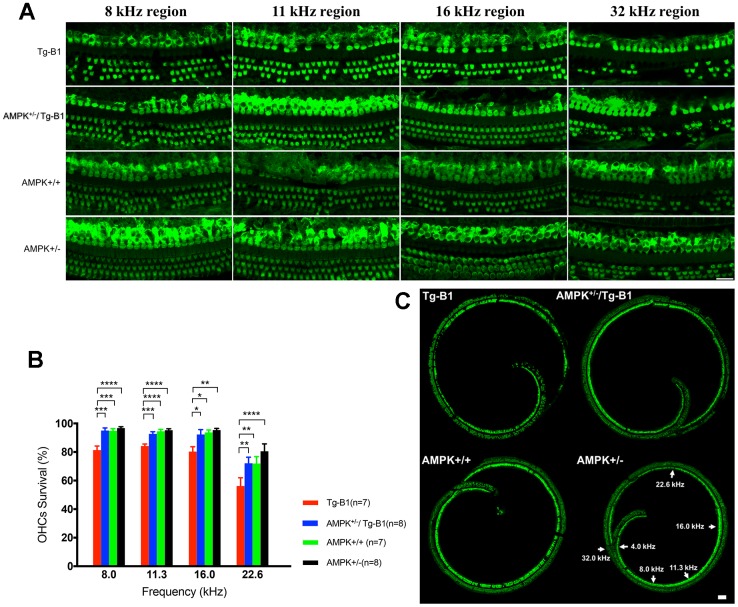
**AMPK KO protects OHCs from damages and losses.** (**A**) Representative immunofluorescent surface preparation images of OHCs from four genotype mice aged 10-12 months were captured at the frequency-specific regions (8, 11.3, 16, and 32 kHz) of the cochleae. The OC was dissected for the staining of hair cells with Myosin7a (green). Scale bar=20 μm. (**B**) Quantification of OHCs survivals in four genotype mice aged 10-12 months. AMPK knockouts increased the number of surviving OHCs in cochlea, differed significantly between Tg-B1 (red bars) and AMPK^+/−^/Tg-B1 (blue bars) mice at 8, 11.3, 16 and 22.6 kHz regions. Values are presented as mean ± SEM and evaluated with two-way ANOVA followed by Bonferroni post-test. (* P<0.05, ** P<0.01, ***P<0.001, **** P<0.0001; n=7 or 8). (**C**) Representative confocal microscopy images from four genotypes of the cochleae. For reference, frequency regions of interest were indicated by the arrowheads. Scale bar=50 μm.

To identify if OHC damage is the source of observed hearing loss in Tg-B1 mice, we quantified the frequency-specific survival of OHCs with MYO7a staining. Surface preparation of cochlear samples at 8, 11.3, 16 and 32 kHz regions (Figure 2A) show Tg-B1 mice have significantly greater OHCs loss than WT controls (Figure 2A and 2B; F _(1,12)_ =21.81, p=0.0005, two-way ANOVA followed by Bonferroni post-test). The number of surviving OHCs in cochlea of AMPK^+/−^/Tg-B1 mice (blue bars) significantly recovered (F_(1,13)_ =17.11, p=0.0012, two-way ANOVA followed by Bonferroni post-test) at frequency regions of 8 kHz (p=0.0001), 11.3 kHz (p=0.0007), 16 kHz (p=0.0212) and 22.6 kHz (p=0.0023, one-way ANOVA followed by Bonferroni post-test) (Figure 2B). These results are in agreement with a previous report that pharmacological treatment with the AMPK inhibitor compound C offers protection from noise-induced OHC loss [17].

### Downregulation of AMPK protects ribbon synapses loss in IHCs and neurodegeneration in spiral ganglion cells, accompanied by improved IHC function

Since the reduction of wave I amplitude suggests that SGN firing is reduced, we then quantified synaptic losses from IHCs. The basal pole of each IHC is surrounded by about a dozen of ribbon synapses, which can be seen in confocal micrographs as closely juxtaposed pairs of fluorescent puncta identifying presynaptic CtBP2 and postsynaptic GluR2. The former is a major component of the synaptic ribbon that promotes the release of glutamate through exocytosis, while the latter is the receptor that glutamate binds and activates [18–20].

**Figure 3:**
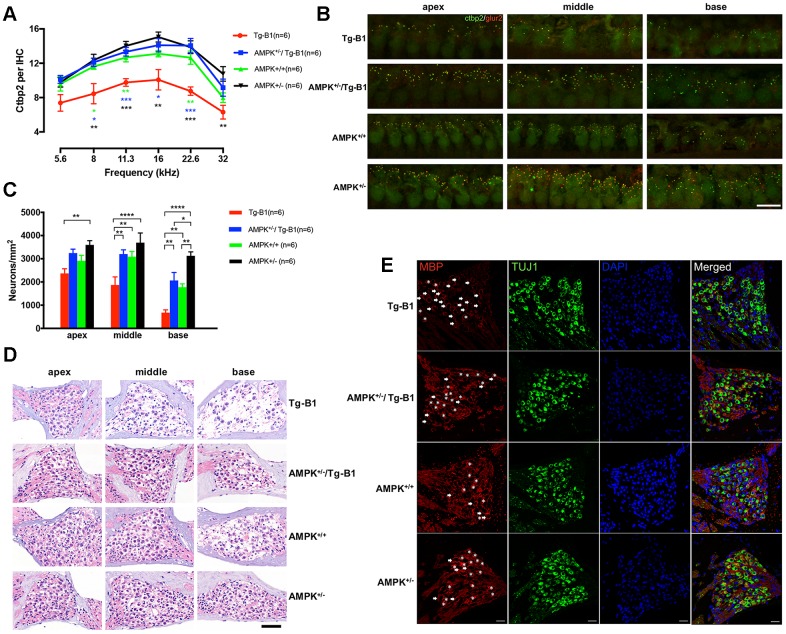
**Downregulation of AMPK protects IHC ribbon synapses and SGNs.** (**A**) Aging Tg-B1 mice showed reduced number of IHC synapses across all frequencies ranged from 5.6 to 32 kHz (F_(1,10)_=25.6, p=0.0005, two-way ANOVA followed by Bonferroni post-test) as compared to wild type controls. Significant increases of ribbon counts in IHCs at 8, 11.3, 16 and 22.6 kHz regions were observed in AMPK^+/−^/Tg-B1 (blue) mice compared to Tg-B1 (red) mice (F_(1,10)_=34.23, p=0.0002, two-way ANOVA followed by Bonferroni post-test) and the former showed almost similar numbers of ribbons to that in WT controls (green). Data are presented as the mean ± SEM, * P<0.05, ** P<0.01, ***P<0.001. n=6. (**B**) Representative z-stack confocal images in the IHC synapse areas from apical, middle and basal cochlear turns in four genotype groups aged 10-12 months showed co-staining in cochlear whole mount preparations with the presynaptic (CtBP2 for RIBEYE, Green puncta) and postsynaptic marker (GluR2, red puncta). CtBP2 in the IHC areas, seen as a cloud of ~0.4-0.6 um puncta, clustered at the basolateral pole. The IHC nuclei were also labeled due to the nuclear expression of CtBP2. Scale Bar=10 μm. (**C**) Statistics of SGN density (Number of SGNs/Area of Rosenthal’s canal) showing the significant SGNs degeneration in Tg-B1 (red bars) mice as compared to WT controls (green bars) (F_(1,10)_ =40.67, p<0.0001, two-way ANOVA followed by Bonferroni post-test), especially in the middle (p=0.0016) and basal turns (p=0.0045) of the cochleae. SGNs survival in AMPK^+/−^/Tg-B1 mice (blue bars) has a remarkable increase compared to Tg-B1 mice (F_(1,10)_=59.99, p<0.0001, two-way ANOVA followed by Bonferroni post-test), especially in the middle (p=0.0017) and basal turns (p=0.0011) of cochleae. (**D**) Representative H&E staining images of SGNs taken from cochleae of aging mice at 10-12 months. The neurons in AMPK KO mice were arranged tightly whereas significant reduction of SGN number occurred in the basal turn of WT controls and more aggravated in the middle and basal turns in Tg-B1 mice. Scale bar=50 μm. (**E**) Representative immunostaining for MBP expression in SGN in four genotype mice. MBP^+^ myelin sheaths (red) enclose type I SGNs (green) in Rosenthal’s canal of the middle turn of the cochlea. SGNs are co-identified with DAPI (blue) and TUJ1(green) staining. The MBP^+^ myelin sheath was considered intact if enveloped more than 80% of the outline of the perikarya. Intact MBP^+^ myelin sheaths are marked by asterisks while broken MBP^+^ myelin sheaths are indicated by arrows. A decline of intact MBP^+^ myelin sheath was found in Tg-B1 mice cochlea. Scale bar=20 μm.

To quantify, we counted the presynaptic ribbons (CtBP2). Compared to wild type controls, aging Tg-B1 mice showed a reduced number of IHC synapses across all frequencies (from 5.6 to 32 kHz, Figure 3A). Notably, IHC synaptic loss in Tg-B1 mice was more substantial in regions of 8-22.6 kHz compared to those in AMPK^+/−^/Tg-B1 mice (Figure 3A), matching the amplitude declines in Tg-B1 mice.

SGN density analysis (Number of SGNs/Area of Rosenthal’s canal) also revealed degeneration in Tg-B1 (red bars) mice (Figure 3C), especially in the middle and basal turns of the cochlea. Survival SGNs in AMPK^+/−^/Tg-B1 mice (blue bars) show a dramatic recovery from Tg-B1 mice (Figure 3C). Accordingly, the SGNs density matched perfectly with the counting results of synaptic ribbons in IHCs (Figure 3A).

The prolonged ABR wave I latencies in Tg-B1 mice indicate that the synaptic transmission and nerve conduction between IHC and SGN are altered. Surrounding the axons of the cochlear nerve fibers, the normal morphology of MBP^+^ myelin sheath encloses the entire SGN and in close contact with the perikaryon [21–23]. In AMPK^+/−^ mice, a separation of myelin sheath enveloping SGNs was occasionally seen (arrow), but without discontinuities (Figure 3E). In Tg-B1 mice, morphological abnormalities in the staining pattern for MBP were often observed in the SGNs, with discontinuous or missing myelin sheaths surrounding SGNs, revealing that partial demyelination occurred in the degenerating SGNs. Meanwhile, the myelinated cell bodies in AMPK^+/−^/Tg-B1 mice appeared to be more intact than that in Tg-B1 mice. The morphological changes in the myelin sheath in Tg-B1 SGNs may account for the underlying source of pathology of prolonged latencies [24] in ABR wave I. In addition, downregulation of AMPK improves overall latency responses, as compared to wild type, suggesting postponed degeneration process of neuronal cells occurred in the cochlea.

**Figure 4:**
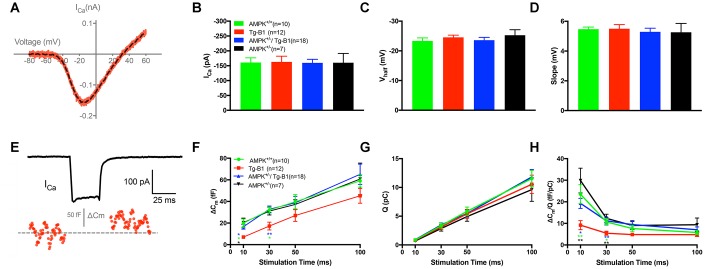
**IHC patch clamp recordings reveal normal calcium current but reduced vesicle release in Tg-B1 mice.** (**A**) Representative trace for voltage-dependent calcium current (I_Ca_) of WT controls mice (red curve) showed I-V relationship of calcium current in IHCs after leakage subtraction and fitted with a double exponential function (dotted line in black). (**B**–**D**) The Ca^2+^ current amplitude (I_Ca_, Panel B), the voltage of half-maximal activation (V_half_, Panel C) and the slope of the calcium activation curve (Panel D) were obtained from the current-voltage relationship fitted with Boltzmann function. No significant differences of I_Ca_ (F_(3,43)_=0.0074, p=0.9991), V_half_ (F_(3,43)_=0.5325, p=0.6624) and Slope of I_Ca_ (F_(3,43)_=0.2187, p=0.8829, one-way ANOVA followed by Bonferroni post-test) were found in IHCs of each group. (**E**) Representative trace of whole-cell membrane capacitance (C_m_) shows measurements of exocytosis of IHC. The depolarization step stimulus induced I_Ca_ and triggered exocytosis (ΔC_m_) in WT. (F-H) Stimulus durations from 10 to 100 ms were applied to examine the release of synaptic vesicles. (**F**): The ΔC_m_ significantly reduced in the Tg-B1 group compared to WT controls for shorter stimuli of 10 ms (p=0.0148) and 30 ms (p=0.0289). AMPK^+/−^/Tg-B1 mice (blue) exhibited significantly larger membrane capacitance change (ΔC_m_) than Tg-B1 mice (red) at stimulus durations of 10 ms (p=0.0463) and 30 ms (p=0.0052, one-way ANOVA followed by Bonferroni post-test). Although presented as a trend, stimulus time longer than 50 ms show no significant difference in ΔC_m_ of all groups. (**G**): Ca^2+^ current charge (Q_Ca_) has no significant difference (F_(3,43)_=0.3077, p=0.8197, two-way ANOVA followed by Bonferroni post-test) for each group for each stimulus duration. (**H**): The ratio of ΔC_m_/Q, which reflects Ca^2+^ efficiency in triggering exocytosis, was significantly lower in IHCs from Tg-B1 mice (red) compared to WT controls (green) for stimulation of 10 ms (p=0.005, one-way ANOVA followed by Fisher’s LSD post-test) and 30 ms (p=0.024, one-way ANOVA followed by Fisher’s LSD post-test), while there was a significant elevation of ΔC_m_/Q for short stimulation of 10 ms (p=0.020, one-way ANOVA followed by Fisher’s LSD post-test) and 30 ms (p=0.005, one-way ANOVA followed by Fisher’s LSD post-test) in AMPK^+/−^/Tg-B1 mice (blue) compared to Tg-B1 group, similar to WT controls and AMPK^+/−^ group (black). All data are presented as means ± SEM; * P<0.05, ** P<0.01; n=10, 12, 18 and 7 for WT, Tg-B1, AMPK^+/−^/Tg-B1 and AMPK^+/−^ group, respectively.

To further evaluate the functional relevance of IHCs and ribbon synapses, we performed whole-cell patch clamp recordings from the apical turn and measured IHC synaptic releases at approximately 8-12 kHz frequencies regions. There is no significant difference in calcium current measurements I_Ca_ (Figure 4B), V_half_ (Figure 4C) and Slope (Figure 4D). To examine both the rapid and sustained synaptic vesicles release, we applied depolarizing pulses (i.e. 0 mV, the potential that gives the maximum Ca^2+^ current) and calculated ΔC_m_ for different stimulus durations from 10 to 100 ms. The ΔC_m_ was significantly reduced in the Tg-B1 group as compared to WT controls, AMPK^+/−^/Tg-B1 and AMPK^+/−^ mice for stimulations of 10 ms and 30 ms (Figure 4F), respectively. Meanwhile, the Ca^2+^ current charge (Q_Ca_) showed no significant difference in four groups at different stimulus durations (Figure 4G). The Ca^2+^ efficiency of triggering exocytosis, quantified as the ratio of ΔC_m_/Q_Ca_, was significantly reduced for stimulation of 10 and 30 ms in the Tg-B1 compared to the WT control group (p=0.0281 and p=0.024, respectively, one-way ANOVA followed by Bonferroni post-test, Figure 4H), suggesting that the persistent decrease of ΔC_m_ for short stimulus duration is due to a decline in the exocytosis efficiency. Furthermore, we found that this decline in the exocytosis efficiency recovered fully in IHCs from the AMPK^+/−^/Tg-B1 group, as ΔC_m_/Q_Ca_ is indistinguishable between this group and the WT control (F_(1,26)_=0.0062, p=0.9381, two-way ANOVA followed by Bonferroni post-test). It is interesting, however, similar changes in ΔC_m_/Q_Ca_ were not found for longer stimulation of 50 ms, likely due to the superlinear Ca^2+^ dependence of exocytosis demonstrated in IHCs [18].

### Downregulation of AMPK rescues stria vascularis dysfunction

Stria vascularis, a densely vascularized epithelium in the lateral wall of the cochlea, generates the potassium-rich endolymph filling the scala media of the cochlea and is responsible for the generation of endocochlear potential (EP). EP supports the electrochemical driving force for sound-induced transduction current through cochlea hair cells [25], playing a vital role in maintaining normal hearing sensitivity [26]. The decline of EP causes the parallel audiometric threshold shift [27].

**Figure 5:**
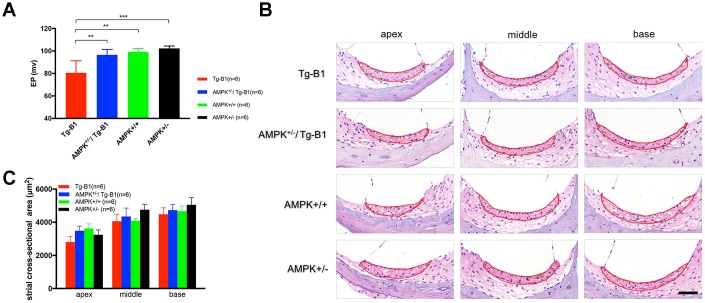
**AMPK KO rescues Endocochlear Potential (EP) in Tg-B1 mice.** (**A**) Profound EP loss in 10-12 months Tg-B1 (red bars) mice was observed as compared to all three other genotypes (Tg-B1 *vs.* WT, p=0.0023; Tg-B1 *vs.* AMPK^+/−^/Tg-B1, p=0.0023; Tg-B1 *vs.* AMPK^+/−^, p=0.0005; one-way ANOVA followed by Bonferroni post-test). * P<0.05, ** P<0.01, ***P<0.001. (**B**) Representative cochlear cross-sections stained with H&E showed gross morphology of stria vascularis in the apical, middle and basal turns of cochleae from four genotypes of mice at 10-12 months. No visible atrophy was found. Scale bar=50 μm. (**C**) The histograms show no significant difference in averaged sectional area of stria vascularis for all four aged genotypes (n=6 for each group; F_(3,20)_=0.8244, p=0.4958, two-way ANOVA followed by Bonferroni post-test).

In the previous report of Tg-B1 mice, EP is reduced along with the progression of hearing loss [12]. In our study, we found that AMPK het-KO mice, both AMPK^+/−^/Tg-B1 and AMPK^+/−^ mice, showed robust EP as compared to Tg-B1 (Figure 5A), implying that downregulation of AMPK protects stria vascularis function. H&E staining of cochlear cross-sections of four genotype mice aged 10-12 months showed relatively normal gross morphology of SV in all three turns (Figure 5B), confirmed by cross-section measurements of SV areas with no atrophy observed in all three turns in Tg-B1 cochlea (Figure 5C).

### Knockouts of AMPKα1 decrease levels of AMPK phosphorylation, attenuate oxidative stress in the inner ear

**Figure 6:**
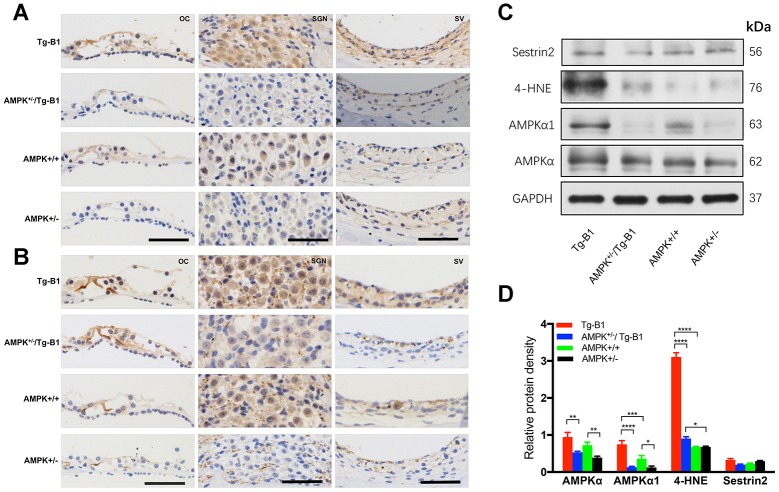
**Expression of AMPK and p-AMPK, quantification of ROS and antioxidant protein in the cochlear tissues.** (**A**–**B**) The representative mid-modiolar immunohistochemistry staining of cross-sections of the cochlea for the expressions of total AMPKα1 and p-AMPKα in three regions of the cochlea: OC (left column), SGN (middle column) and SV (right column). Increased DAB-stained immunolabeling of AMPKα1 and p-AMPKα (brown) in the cytosol and nuclei of OHCs, IHCs, OC, SGNs, basal cells of the SV were observed in the cochlear sections of Tg-B1 mice than those in AMPK^+/−^/Tg-B1 and WT mice. There was strong immunolabeling for p-AMPKα in the OC, SGNs, and SV of Tg-B1 mice, while AMPK^+/−^ showed the weakest immunolabeling signals. Scale bar=50 μm. (**C**) Western blot using sensory epithelium tissues from 10-12 months mice displayed significant alteration in band density for total AMPKα, AMPKα1, and 4-HNE in the cochleae between Tg-B1 and AMPK^+/−^/Tg-B1 mice, but no significant difference in Sestrin2 expression, the antioxidant protein. GAPDH served as the loading control. (**D**) Histograms (mean ± SEM) represent relative density values normalized to GAPDH. Blotting results of AMPKα1 showed knockouts of AMPKα1 in Tg-B1 mice significantly decreased the AMPKα1 (p<0.0001) expression in the inner ear. Western blot analyses of 4-HNE expression in Tg-B1 mice cochleae were significantly higher than WT controls (p<0.0001) and KO mice (p<0.0001). Experiments were performed in triplicate, and p-values were determined by one-way ANOVA followed by Bonferroni post-test.

AMPK activation and its response to alterations in intracellular metabolic pathways can be directly regulated by cellular redox status [28–30]. The level of p-AMPKα may therefore positively correlate with ROS levels. To confirm AMPK expression and its phosphorylation in the het-KO mice cochlear tissues, cross-sections were stained with immunohistochemistry approach. KO mice cochlea showed notably decreased DAB-stained immunolabeling for either AMPKα1 (Figure 6A) or p-AMPKα (Figure 6B) in the SGN, SV and OC compared to those in Tg-B1 mice, in agreement with the blotting outcomes (Figure 6C).

The average level ratio of total AMPKα in the Tg-B1, AMPK^+/−^/Tg-B1, AMPK^+/+^ and AMPK^+/−^ mice are 0.9459, 0.5306, 0.7241, 0.3906; The average level ratio of AMPKα1 in the four genotypes are 0.7491, 0.1312, 0.3573, 0.1248, respectively. These results confirmed that genetic knockouts of AMPKα1 in Tg-B1 mice and wild type controls significantly decreased the levels of AMPKα1 (Figure 6D) expressed in the inner ear. Additionally, levels of oxidative stress markers 4-hydroxynonenal (4-HNE) in Tg-B1 mice cochleae were significantly higher than wild type controls and ROS expressed in the cochleae of AMPK^+/−^/Tg-B1 mice were significantly lower than that of Tg-B1 mice (Figure 6D), indicating that decrease of AMPK resulted in decreased oxidative stress in the cochlea. However, expression of Sestrin2, a key inhibitor of TORC1 activation, remains unchanged (p>0.05, one-way ANOVA followed by Bonferroni post-test).

### Genetic reduction of AMPK decreases the ROS-induced apoptotic signaling in inner ear, providing rescue from deafness

To investigate the molecular mechanism underlying the rescue of hearing loss in Tg-B1 mice, we analyzed the expression of proteins that related to apoptosis and autophagy in cochlear tissues. Bax acts as a promoter of apoptosis, mediating the release of pro-apoptotic factors from the mitochondrial intermembrane space into the cytoplasm [31]. Bcl-2, an anti-apoptotic protein, counters the pro-apoptotic properties of Bax, has been proved to effectively prevent Bax from oligomerization in the mitochondrial outer membrane [32]. Proteins that promote or inhibit apoptosis interact and the ratio between the two (Bcl-2/Bax) determines the cellular fate [33]. Cell survival can be threatened by declined expression of Bcl-2 and a simultaneous increase in Bax levels [31].

**Figure 7:**
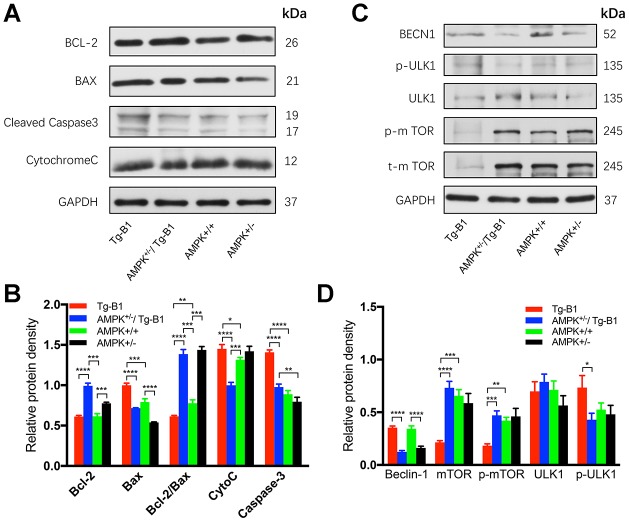
**AMPK knockout interrupts and decreases apoptosis in the cochlea.** (**A**) Immunoblots analyses of the four genotype mice aged 10-12 months show proteins from the cochleae related to the apoptotic pathway, including Bcl-2, Bax, Caspase-3, and Cytochrome C. GAPDH serves as the loading control. (**B**) The histograms summarized the expression levels of proteins related to the apoptosis pathway. The apoptosis signaling (cleaved caspase-3) in Tg-B1 mice was significantly stronger than AMPK KO mice and WT controls (Tg-B1 *vs.* AMPK^+/−^/Tg-B1, p<0.0001; Tg-B1 *vs.* WT, p<0.0001; AMPK^+/−^/Tg-B1 *vs.* WT, p=0.1807). The expression of pro-apoptotic protein (Bax) in Tg-B1 mice is significantly higher than the other three groups (Tg-B1 *vs.* AMPK^+/−^/Tg-B1, p<0.0001; Tg-B1 *vs.* WT, p<0.001; Tg-B1 *vs.* AMPK^+/−^, p<0.0001; WT *vs.* AMPK^+/−^, p<0.0001; AMPK^+/−^/Tg-B1 *vs.* AMPK^+/−^, p=0.0001). Bcl-2/Bax ratio in the Tg-B1 group is significantly lower than the other three groups (Tg-B1 *vs.* AMPK^+/−^/Tg-B1, p<0.0001; Tg-B1 *vs.* WT, p=0.0072; Tg-B1 *vs.* AMPK^+/−^, p<0.0001), so does the wild type to AMPK^+/−^ group (p=0.008). Experiments were performed in triplicate, and p-values were determined by one-way ANOVA followed by Bonferroni post-test. n=3 per group. (**C**) Western blot results show changes in autophagy-related proteins in the cochleae of aging mice. There is a remarkable decline of mTOR signaling (Tg-B1 *vs.* AMPK^+/−^/Tg-B1, p<0.0001; Tg-B1 *vs.* WT, p=0.0001) and more Beclin-1 (Tg-B1 *vs.* AMPK^+/−^/Tg-B1, p<0.0001, one-way ANOVA followed by Bonferroni post-test) expressed in the cochleae of Tg-B1 mice. (**D**) The histograms of western blot analyses show knockouts of AMPK relieve the ROS-induced autophagic stress in Tg-B1 mice. Analysis performed by using Image J software and one-way ANOVA followed by Bonferroni post-test. * P<0.05, ** P<0.01, ***P<0.001, **** P<0.0001; n=3 per group.

We analyzed the expression of Bax, Bcl-2, Cytochrome C and cleaved caspase-3 proteins in auditory sensory cells (Figure 7A and 7B), and observed in the AMPK^+/−^/Tg-B1 group, the level of Bax protein decreased by 28% relative to Tg-B1 group (p<0.0001). Expression of Bax (the pro-apoptotic Bcl-2 protein) is upregulated in an AMPK-dependent manner in Tg-B1 group, indicating Bax expression plays a crucial role in AMPK-mediated apoptosis as shown by het-knockouts of AMPKα1. Quantitative analysis of Bcl-2/Bax ratio (anti/pro-apoptotic) show significant differences in the four genotype groups (F_(3,8)_=312.3, p<0.0001, one-way ANOVA followed by Bonferroni post-test, Figure 7B). Downregulation of AMPK promoted a significant increase in the Bcl-2/Bax protein ratio, implying less pro-apoptotic signaling and more auditory cell survival in the cochlea of AMPK KO mice, matched perfectly with the morphological results of OHCs and SGNs (Figure 2B and 3C). Thereby, het-knockouts of AMPKα1 in Tg-B1 mice could block the AMPK-mediated apoptotic pathway and ultimately reduce the expression of cleaved caspase-3 (Figure 7B), in line with the results of the Bcl-2/Bax ratio. However, KO mice showed the similar level of cleaved-caspase-3 as compared to WT controls (p=0.1572, one-way ANOVA followed by Bonferroni post-test), seemed incompatible with Bcl-2/Bax ratio, indicating there may be other factors at play. Regarding caspase-3, we point out that this is cleaved caspase-3, the active form of this caspase, and that total levels of caspase-3 was found unchanged in the previous instances [12, 14].

Furthermore, the genetic reduction of AMPK can relieve autophagic stress due to the decreased ROS in cochlea via activation of mTOR, the inhibitor of autophagy. As shown in the blotting result, little or no mTOR was observed in Tg-B1 group (Figure 7C and 7D), resulting in autophagy by activation of ULK1 (Figure 7D) that may help to relieve oxidative stress in cochleae. Levels of the autophagy marker Beclin-1 expressed in Tg-B1 mice are significantly higher than that in the AMPK^+/−^/Tg-B1 group (p<0.0001, one-way ANOVA followed by Bonferroni post-test) (Figure 7D). However, the mechanism of that mTOR exhibits a dramatic decline in the cochleae of Tg-B1 mice remains to be explored.

### Genetic knockouts of AMPKα1 attenuate noise-induced hearing loss (NIHL) and protect the inner ear against synaptic injury

Previous researches reported that the reduction of ROS activated AMPK rescued hearing loss in aging Tg-B1 mice [12, 14]. The inhibition of AMPK via siRNA or compound C can also attenuate noise-induced loss of hair cells and cochlear synaptopathy [17]. Based on these findings, we here explore if the rescue effect extends to normal animal, thereby AMPK serves as a novel route to prevent NIHL, and perhaps age-related hearing loss in general.

**Figure 8:**
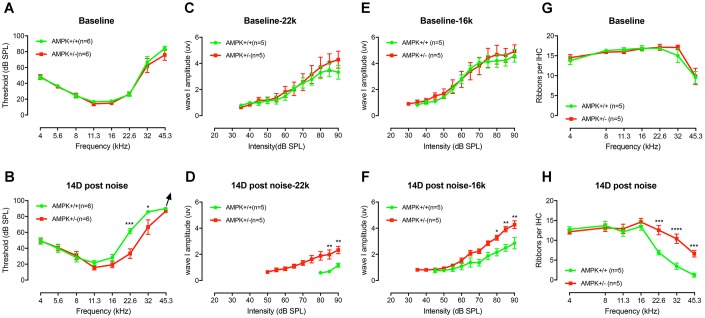
**AMPK KO protects the noise-induced hearing loss and synaptopathy.** (**A**) The baseline ABR thresholds for the two genotype groups were similar at the age of 1-2 months (F_(1,10)_=0.0095, p=0.3419). (**B**) An episode of two-hour, 106 dB SPL noise exposure induces significant threshold shifts at 22.6 kHz and 32 kHz 1 d after exposure (data not shown). Complete threshold recovery were found in AMPK^+/−^ group at 14d (F_(1,10)_=3.455, p=0.0927) but not the WT (F_(1,10)_=15.22, p=0.0030). Significant difference in thresholds between both genotypes on D14 post-exposure (F_(1,10)_=5.776, p=0.0371), for 22.6 kHz (p=0.0001) and 32 kHz (p=0.0214), respectively. (**C**–**F**) ABR wave I amplitudes, evoked by suprathreshold tones at 16 (**C**) and 22.6 kHz (**E**), have no significant difference before noise exposure (for 16 kHz, F_(1,8)_=0.05484, p=0.8207 and for 22.6 kHz, F_(1,8)_=0.2944, p=0.6022). WT mice suffer more severe ABR wave I amplitude reduction at 16 (**F**) and 22.6 kHz (**D**) than AMPK KO mice 14 days after noise exposure (for 16 kHz, F_(1,8)_=17.85, p=0.0029 and for 22.6 kHz, F_(1,8)_=14.43, p=0.0052). 14 days after noise, the noise-induced decrease in wave I amplitudes in wild type group was significantly elevated at 22.6 kHz (from 3.32 ± 0.53 μV to 1.17 ± 0.15 μV) and at 16 kHz (from 4.58 ± 0.48 μV to 2.85 ± 0.43 μV), whereas in AMPK^+/−^ group, the wave I amplitudes at 22.6 kHz (from 4.29 ± 0.64 μV to 2.33 ± 0.28 μV) and at 16 kHz (from 4.93 ± 0.50 μV to 4.27 ± 0.30 μV) had little change following acoustic trauma. (**G**) Numbers of CtBP2 in IHCs from both groups before noise exposure show no significant difference between the two genotype groups (F_(1,8)_=0.3357, p=0.5783). (**H**) At 14 days post-exposure, wild type mice suffer more loss of CtBP2 in the region of 22.6, 32, and 45.3 kHz than AMPK KO mice. (KO *vs.* WT, F_(1,8)_=13.24, p=0.0066; for 22.6 kHz, p=0.0004; for 32 kHz, p<0.0001 and for 45.3 kHz, p=0.0008). Two-way ANOVA followed by Bonferroni post-test was applied in the statistical analysis of the figure. n=5 or 6 for each group. Data presented as mean ± SEM; * P<0.05, ** P<0.01, ***P<0.001, **** P<0.0001.

Although there was no significant difference in the baseline threshold (Figure 8A), amplitude of ABR wave I (Figure 8C and 8E), and CtBP2 counts per IHC (Figure 8G) between WT controls and AMPK KO mice at age of 1-2 months, after one episode of 2 hours, 106dB noise exposure, we found significant differences in threshold recovery between the two genotypes (Figure 8B). Specifically, in the AMPK het-KO group, 106 dB noise-induced temporary threshold shift recovered to the baseline levels after 14 days, whereas a residual moderately elevated hearing thresholds at high frequencies (22.6 kHz and 32 kHz) in WT controls remained (Figure 8A and 8B). In addition, the number of presynaptic ribbons per IHC in WT group was significantly reduced (F_(1,8)_=116.4, p<0.0001), coinciding with the ABR threshold shift in high frequency region (Figure 8H).

It has been known that synaptic losses after noise exposure can contribute to reductions in supra-threshold amplitudes of ABR wave I, a phenomenon termed synaptopathy [34, 35]. We analyzed the amplitudes of ABR wave I in noise affected frequencies (>8 kHz). In contrast to AMPK het-KO mice, WT littermate controls showed significant declines of wave I amplitudes at 22.6 kHz (Figure 8D) and 16 kHz (Figure 8F) at 14 days post-exposure. ABR wave I amplitudes of WT and AMPK^+/−^ mice elicited by 90 dB SPL tones at 22.6 kHz were significantly reduced by 65% and 45% following acoustic trauma, respectively. Likewise, ABR wave I amplitude elicited by 90 dB SPL tones at 16 kHz were significantly attenuated by 38% and 13% in the two groups after noise exposure, respectively, demonstrate that AMPK het-KO exerts a protective effect on NIHL.

## DISCUSSION

In a previous report, Tg-B1 was generated as the first animal model for mitochondrial dysfunction that recapitulates some aspects of human A1555G mutation [14]. Progressive hearing loss was first observed, then in a follow-up study, proved to be associated with the EP change [12]. It is further demonstrated that the genetic downregulation of AMPK rescued or delayed the hearing loss in general. There were also tissue-specific phenotypes in Tg-B1 mice, such as prolonged ABR wave I latency, implying that either IHC or SGN functions are affected [12]. In this study, we explore the tissue-specific consequence of downregulation in AMPK signaling and its impact on auditory function. The results in this study also extend to age-related hearing loss in general in that AMPK can serve as a therapeutic target to prevent the progression of hearing loss, either due to age or accumulation of environmental insult.

### Consideration of auditory phenotypes in the mitochondrial deafness mouse model

As mentioned above, progressive hearing loss was observed in Tg-B1 mice, accompanied by decreased auditory brainstem response peak I amplitude and prolonged wave I latency. Meanwhile, we can conclude from the severe OHC loss, IHC ribbon synapses reductions and spiral ganglion cell death in aging Tg-B1 mice that sensory hair cells and SGNs were compromised in the pathogenesis of hearing loss in Tg-B1 mice, conspiring to promote the overall elevations in ABR thresholds. The death of OHCs was a source of the observed auditory phenotype of threshold elevation; while the loss of synaptic connections matched with the overall amplitude declines. The previous report has observed that TFB1M overexpression in the cochlea induces long-term changes in OHC voltage responsiveness [12] with potential negative impacts on cochlear amplification [36]. Besides, demyelination in MBP impaired auditory nerve firing activity and the altered neuroanatomical features in SGN were in line with the prolonged latencies in Tg-B1 mice. IHC function was also altered in terms of the declined exocytosis of synaptic vesicles release in IHCs, probably due to the mobilization of synaptic vesicles for exocytosis and recycling which is highly dependent on mitochondrial energy supply [37, 38].

In respect of SV, Tg-B1 mice showed reduced EP as compared to WT controls, indicative of significant SV dysfunction. EP is essentially a K^+^ equilibrium potential, the primary charge carrier for sensory transduction [39], and serves as the energy source for the transduction current that drives the electromotility of the OHCs [25, 40, 41], namely the cochlear amplifier. EP reduction can be a result of overloaded ROS to the stria or spiral ligament [42–44] or increased endolymph leakage into the OC [45]. Additionally, a decline in EP corresponds to decreased Na-K-ATPase (NKA) activity [46]. Disturbance of cochlea homeostasis can result in both syndromic and non-syndromic forms of hereditary hearing loss [25]. The EP reduction in aging Tg-B1 mice may result from mitochondrial and vascular dysfunction, albeit no obvious signs of strial atrophy was observed in SV of Tg-B1 mice.

### Consideration of pathogenic mechanism

**Figure 9:**
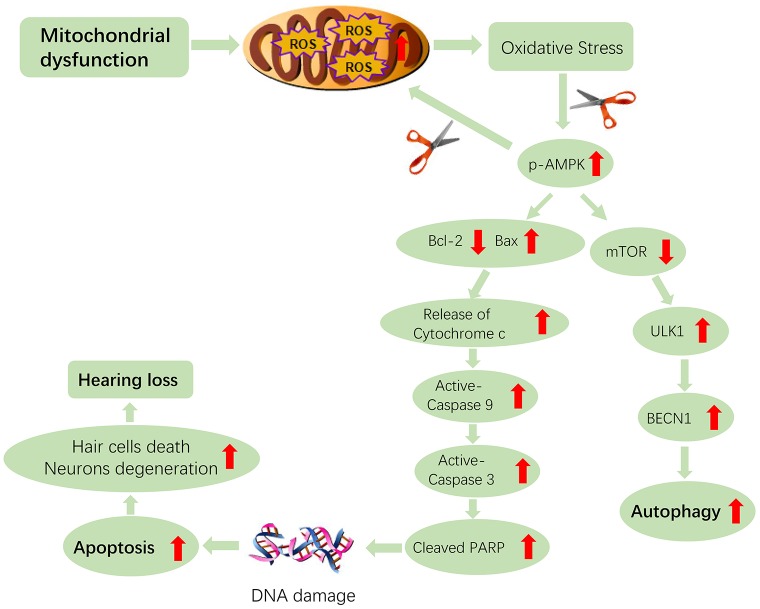
**Hypothetical scheme of molecular and cellular events in the cochleae of aging Tg-B1 mice leading to accelerated hearing loss.** The aberrant activation of AMPK induces the ROS-AMPK-Bcl2 apoptotic pathway in cochleae, resulting in the increased sensory hair cell loss and SGN death. The “scissor” in the figure represents the knockouts of the AMPK pathway. The conjectured downregulation of AMPK could attenuate the apoptotic signaling and ROS accumulation in auditory cells, which accounts for rescue of hearing loss in Tg-B1 mice.

A previous report revealed that mitochondrial dysfunction and oxidative stress are the primary pathogenic mechanisms of hearing loss in Tg-B1 mice [14]. Likewise, it can be inferred from our blotting results that the auditory pathologies in aging Tg-B1 mice were due to oxidative stress occurring in the mitochondria of hair cells, SGNs and epithelial cells of the SV, triggered by activation of AMPK and further pro-apoptotic signaling (Figure 9). Our study confirmed AMPK activation was involved in hearing loss and apoptosis induction. Overproduction of ROS in auditory sensory cells, mediated by activation of AMPK, triggered Bcl-2/Bax-dependent apoptosis of auditory cells, can make them more susceptible to apoptotic conditions.

Sestrin-2 (Sesn2), a member of the stress-responsive proteins, is known to play a protective role in regulating cell growth and viability, against oxidative stress and age-related pathologies [47]. Furthermore, previous studies have shown that Sesn2 negatively regulates mTORC1 via AMPK and thus attenuates ROS accumulation [48], which may reduce Endoplasmic Reticulum stress or induce autophagy, promote survival of hair cells after aminoglycoside exposure [49]. In short, Sesn2/AMPK/mTOR signaling plays a central role in the regulation of redox homeostasis related balance between survival and apoptosis in sensory hair cells [47]. However, no difference of Sesn2 expression existed in cochlear tissues of Tg-B1 mice, indicating that oxidative stress did not compromise the antioxidant protein Sesn2 and that AMPK signaling could not reciprocally react on it, therefore exclude Sesn2 as a player in response to increased ROS in Tg-B1 cochleae.

### Consideration of rescue machinery

The observed auditory phenotypes in Tg-B1 mice were remarkably rescued by genetic knockouts of AMPK: showing robust ABR wave I, EP and IHC function; as well as SGNs, IHC synapses, and OHC survivals. Accordingly, the downregulation of AMPK protects the integrity of sensory hair cells, ribbon synapses, SGNs, as well as stabilized EP, which jointly contribute to the rescued auditory function.

Bcl-2(B-cell lymphoma 2) family members play a crucial role in modulating apoptosis, either to inhibit or to trigger cell death through the intrinsic pathway of apoptosis [50]. It has been reported that AMPK can upregulate pro-apoptotic proteins of Bcl-2 family such as Bim (Bcl-2 interacting mediator of cell death) and Bax (Bcl2-associated X protein) [51–53], which translocate to the mitochondria, then induce Cytochrome C release into the cytosol and permeabilization of the outer mitochondrial membrane (OMM), followed by downstream caspases activation and subsequently trigger cell death, along with biochemical and morphological changes of apoptosis or necrosis, which is decided by cellular ATP level and redox changes interfering with caspase activity [50]. The released pro-apoptotic factors trigger the formation of the apoptosome, caspase-9 proteolysis and activation of caspase-3 which cleaves many protein substrates, ultimately leading to cell death [31]. Apoptosis-dependent cell death pathway is biochemically characterized by protein cleavage, apoptotic chromatin condensation, protein cross-linking, DNA fragmentation, phagocytosis and cell death [50, 54, 55]. Accordingly, that may be the process of apoptosis occurring in auditory cells (Figure 9).

As observed in our study, by downregulation of AMPK signaling, ROS-induced oxidative stress in cochlear tissues was significantly attenuated and followed by decreased apoptosis induction through blocking-up the ROS-AMPK-Bcl2/Bax pathway, then reduced the AMPK-mediated apoptosis of spiral ganglion cells and sensory hair cells in the inner ears, accounting for its overall efficacy in rescue hearing loss in Tg-B1 mice. Therefore, AMPK is key to premature senescence in auditory cells in an apoptosis-dependent manner. It is worth mentioning that previous research findings have demonstrated AMPK serves as a pro-apoptotic molecule mediating apoptosis via diverse pathways. C-Jun N-terminal kinase (JNK) pathway is viewed as a mediator of AMPK-induced apoptosis [56–58]. AMPK can also regulate and stabilize tumor suppressor transcription factor p53, which is also an apoptosis-inducer [52, 59, 60].

Besides, AMPK activation promotes nonselective phagocytosis [61], along with enhancing autophagy induction [62]. Autophagy is an intrinsic cellular process that protects against non-syndromic hearing loss (NSHL) by attenuating oxidative stress [63]. In particular, AMPK can improve mitochondrial autophagy originated from reactive oxygen damage [64], for instance, AMPK has been identified as a sensor of oxidative stress to elicit neuronal atrophy in Huntington’s disease [15] and regulate neurodegenerative diseases via mitochondrial autophagy in Alzheimer’s disease [62]. ROS-activated AMPK is a key regulator of autophagy via inhibiting mammalian target of rapamycin (mTOR), which is known as the growth factor-regulated and nutrient-sensing kinase, along with the autophagy-initiating kinase ULK1 (unc-51-like kinase1, a homologue of yeast and fly ATG1) joint interaction to regulate the autophagic machinery [65–68], exerting a pro-survival effect rather than apoptosis. In fact, it is conceivable that the pro-apoptotic or anti-apoptotic effects of AMPK vary with the AMPK isoform [58]. It can be inferred that excessive ROS in Tg-B1 mice overwhelms the beneficial potential of autophagy in OHCs and SGNs, leading to the observed phenotypes of auditory cell death and apoptosis.

Consequently, further studies remain to be conducted to investigate how apoptosis and autophagy impact auditory cellular function and contribute to pathology underlying hearing impairments.

### Consideration of noise susceptibility

It is known that acoustic overstimulation leads to sustained AMPK activation, resulting in cochlear synaptopathy with subsequent NIHL [17], consistent with the declined auditory phenotypes in WT controls after noise exposure in our study. Disruption of AMPK signaling through transient inhibition of AMPK activation, either via siRNA silencing or pharmacological inhibitor, can prevent the noise-induced imbalance of AMPKα signaling, including the reduction of ABR wave I amplitudes, loss of IHC synaptic ribbons and OHCs death [17]. The previous study showed that genetic knockouts of AMPK rescued the Tg-B1 phenotype [12]. We further prove that the reduction of AMPK, in general, can protect against the hearing loss that resulted from acoustic trauma. On the other hand, Foller et al. put forward that permanent genetic knockout of AMPKα1 increased noise vulnerability [69], and its underlying mechanism may be involved in alternatives in BK channels. AMPK is an effective regulator of BK channels in the protection against NIHL [69], and lack of BK channels increases susceptibility to acoustic overstimulation [70, 71].

Taken together, the main finding of this study is that AMPK mediates the hearing loss in Tg-B1 mice via ROS-induced apoptotic signaling which involves Bcl-2 family proteins in the cochlea, unraveling a novel ROS-AMPK-Bcl2 pathway in the regulation of cochlear apoptosis. As it manifested attenuated oxidative stress and apoptotic cell death in auditory sensory cells through reducing AMPK signaling, we propose that downregulation of AMPK (such as inhibition or genetic knockouts) could, therefore, be essential for auditory rescue and treatment of ROS-induced hearing loss, either due to age or cumulative environmental insult.

## MATERIALS AND METHODS

### Animals care and breeding

The animal breeding protocols were in compliance with the guidelines for experimental animal welfares of Ninth People’s Hospital, Shanghai Jiao Tong University School of Medicine. All experimental procedures were approved by the Animal Ethics Committee of the Ninth People’s Hospital of Shanghai Jiao Tong University School of Medicine, Shanghai, China.

Mice were kept in a 12-hour light-dark cycle, with the humidity of ~50% and room temperature set at 22°C, with low ambient acoustical noise (<50dB SPL throughout 1-100 kHz). All the animals were housed in cleaned corncob beddings and given free access to food and water.

Tg-B1 mice were cross-bred with AMPKα1 het-knockout (AMPK^+/−^) mice (both are of C57BL/6J background), to generate heterozygous for AMPK that over-express Tg-B1 (AMPK^+/−^/Tg-B1). Then the AMPK^+/−^/Tg-B1 mice were bred to AMPK^+/+^ mice results in four genotypes: Tg-B1, AMPK^+/−^/Tg-B1 (Tg-B1 with reduced AMPK signaling), AMPK^+/+^ (wild type C57BL/6J) and AMPK^+/−^. We chose age-matched littermates of the four genotypes to explore the auditory phenotype and mechanisms of premature hearing loss.

### Genotyping and DNA sequencing

All the experimental animals were genotyped from DNA obtained by a tail clipping upon weaning. The following primers were used for PCR procedure:

Tg-mtTFB1 genotyping: mtTFB1 exon F: 5’-ATGGCTGCCTCGGGGAAGCT -3’; mtTFB1 intron R1: 5’-AACTGCAAACTCAGGGCTGT -3’; mtTFB1 exon R2: 5’-AACCCTGGGATAAAGCGAGTGTC -3’.

AMPKα1 het-KO genotyping: alpha1 F: 5’-AGCCGACTTTGGTAAGGATG -3’; alpha1 R: 5’-CCCACTTTCCATTTTCTCCA -3’.

The PCR cycling parameters were set as follows: 94°C for 15 minutes; 35 cycles of 94°C for 30 seconds, 60°C for 1 minute 30 seconds and 72°C for 1 minute 30 seconds; 72°C for 10 minutes; 24°C hold. The amplified products of PCR were separated on 3% Agarose gel. The expected band size for wild type and AMPK α1 het-KO allele were 450 and 350 bps, for endogenous B1 and transgenic B1 were 510 and 281bps, respectively.

### Auditory Brainstem Responses (ABR) testing and analysis

Mice were anesthetized by an initial intraperitoneal (IP) injection of 480 mg/kg chloral hydrate (Sigma Aldrich-Fluka, St. Louis, MO, USA) and placed in a double-wall sound isolation chamber. Body temperature was maintained at 37°C with an isothermal pad (Harvard Apparatus, 55-7020). Supplemental doses of 120 mg/kg were given as needed.

For ABR recordings, three subdermal needle electrodes were placed at the vertex (active electrode), right infra-aural mastoid (reference electrode) and left shoulder (ground electrode), respectively. Stimulus generation and recordings were accomplished by using a TDT RZ6 workstation (Tucker–Davis Technologies, Alachua, FL, USA). A short tone pip of 3 ms duration with 1 ms rise/fall time was delivered free field through a speaker positioned 10cm from the ears, at a rate of 20 stimuli per second. ABR recordings were conducted by series of short tone pips (at 4, 5.6, 8, 11.3, 16, 22.6, 32, and 45.3 kHz, decreased from 90 to 0 dB SPL, in 5-dB steps). Waveforms were averaged 400 trials for each stimulus level and each frequency by using the BioSigRZ software.

Thresholds were defined as the minimal sound levels where the ABR waves were evoked. All the ABR testing and threshold assessments were conducted by the same experienced experimenter who was “blind” to the genotypes of mice to avoid subjective bias. Near thresholds, the recordings were repeated twice to confirm the findings. Analysis of ABR waves were measured post hoc. Latencies were defined as the time of the onset of the stimulus to the peak of wave I; while amplitudes of ABR wave I were estimated by averaging the ΔV of both sides of the peak.

### Endocochlear potential recordings

Mice were anesthetized by IP injection of a mixture of 20 mg/kg xylazine and 100 mg/kg ketamine. Body temperature was maintained at 37°C on a thermostatically-controlled operating table (Harvard Apparatus, 73-3771), where a mouse was kept in a supine position through a head holder (MA-6N, Narishige, Tokyo, Japan). A tracheotomy was performed to allow unobstructed breathing. The round window of Cochlea was then exposed through a ventral approach. A glass microelectrode (1B150F-4; World Precision Instruments, Sarasota, FL, USA) filled with 3 M KCI with the resistance of 12–20 MΩ was mounted on a motorized manipulator (IVM Single, Scientifica Limited, East Sussex, UK) as recording electrode. Ground Ag-AgCl wire was placed into the neck musculature. An Axopatch 200B amplifier (Molecular Devices, LLC., San Jose, CA, USA) with an Axon Digidata 1550B and interfaced by software pClamp (version 10.6, Molecular Devices, LLC., San Jose, CA, USA) were used for the current-clamp recording of EP. The potential was zeroed as the baseline after the microelectrode advanced through the round window membrane into scala tympani. The microelectrode was then advanced into the scala media through the basilar membrane to record EP. As confirmation, recovery of voltage from EP needs to be observed by first withdraw the recording electrode from the endolymph, followed by continuous advance through scala vestibuli.

### Immunohistochemistry

### Cochlear tissue preparation

After overdosing the animals, cochleae were harvested from dissected temporal bones. A small hole was punctured at the apical cochlear bone and 4% paraformaldehyde in 0.1 mmol/L phosphate-buffered saline(PBS) was then quickly perfused through the round window and oval window, and then leave overnight at 4 °C. Followed by decalcification with 10% EDTA for 4-5 days.

For cochlear surface preparation, after removing the softened otic capsule, stria vascularis, Reissner’s membrane and tectorial membrane were exposed under a dissecting microscope. The basilar membrane was then dissected and cut into three segments: apical, middle and basal turns of the OC.

For frozen cochlear sections, each decalcified cochlea was placed into the 15% and 30% sucrose solution successively until the tissue sank. Then the dehydrated cochlea was embedded in degassed OCT compound (4583, Tissue-Tek, Torrance, CA, USA) at -20°C, frozen mid-modiolar sections (10μm) cut by a freezing microtome (CM3050S, Leica Biosystems Inc., Buffalo Grove, IL, USA) were collected on glass slides for later fluorescent staining, stored at-20°C before use.

For cochlear paraffin sections, following decalcification, each cochlea underwent gradient dehydration of ethanol and then embedded with paraffin. Paraffin-embedded specimens were cut through the modiolus with 4 μm thickness, and we selected morphologically intact slices to conduct H&E staining.

### Immunofluorescence staining and confocal imaging

The cochlear sensory epithelium was permeabilized in 1% Triton X-100 solution for 30 min and blocked with 5% goat serum in PBS for 60min at room temperature. Then the tissues were immersed in the following primary antibodies at 4 °C overnight: polyclonal rabbit anti-Myosin VIIa (25-6790, Proteus BioSciences, Ramona, CA, USA) at 1:400, mouse (IgG1) anti-CtBP2 (612044, BD Biosciences, Becton, Dickinson and Company, Minneapolis, MN, USA) at 1:200 and monoclonal mouse anti-Glutamate Receptor2 IgG_2a_ (MAB397, Millipore, Darmstadt, Germany) at 1:400. After being washed three times in PBS for totally 30 minutes, tissues were incubated in the secondary antibodies at a concentration of 1:400 for 2-3 hours at room temperature in darkness: Alexa Fluor 555-conjugated donkey anti-rabbit IgG (A-31572), Alexa Fluor 633-conjugated goat anti-mouse IgG1 (A-21126) and Alexa Fluor 488-conjugated goat anti-mouse IgG_2a_ (A-21131), all provided by Invitrogen (Thermo Fisher Scientific, Inc., Waltham, MA, USA). After 3 times of washing with PBS, specimens were rinsed in anti-fade reagent (P10144, Thermo Fisher Scientific, Inc., Waltham, MA, USA) and mounted on a slide. Mid-modiolar cryosections were co-labeled with antibodies against Tuj1 (801202, Biolegend, San Diego, CA, USA) at 1:200 and Myelin Basic Protein (MBP) (ab62631, Abcam, Cambridge, England, UK) at 1:100 as above.

Immunolabeled images were taken using a Zeiss LSM 880 laser scanning confocal microscope (Carl Zeiss Microscopy, Jena, Germany). The staining intensity was measured by using Image J software (Wayne Rasband, National Institutes of Health, Bethesda, MD, USA)

### Immunohistochemistry for cochlear paraffin sections

Cochlear paraffin sections were deparaffinized with xylene and rehydrated in ethanol at graded concentrations. Rinsed in 3% hydrogen peroxide for 10 minutes and block solution for 30 minutes at room temperature. Then immersed in the following primary antibodies in a wet box at 4 °C overnight: anti-p-AMPKα (2535, Cell Signaling Technology Inc., Beverly, MA, USA) at 1:100, anti-AMPKα1 (ab32047, Abcam, Cambridge, England, UK) at 1:200 and anti-AMPKα2 (ab3760, Abcam, Cambridge, England, UK) at 1:100, respectively. Then incubated in the secondary antibodies HRP-labeled goat anti-rabbit IgG (A0208, Beyotime, Shanghai, CN) for 30 minutes in darkness at room temperature. The peroxidase reaction was visualized by using diaminobenzidine (DAB) reagent. The slides were finally dehydrated, cleared and mounted with coverslips.

### Hematoxylin and Eosin staining (H&E staining)

To quantify SGN survival and histopathology, cochlear slices were stained with H&E. Firstly, cochlear paraffin sections were routinely deparaffinized in xylene and rehydrated in ethanol. Secondly, immerse sections in hematoxylin for 5 minutes and wash out with tap water. Then immerse sections in eosin for 3 minutes and washed with tap water for 10 minutes. Dehydrate sections in ascending concentrations of alcohol solutions and clear with xylene. Finally, mount coverslip onto a glass slide with Permount. Nuclei and other basophilic structures were blue while cytoplasm and acidophilic structures were light to dark red.

### Quantification of OHCs, CtBP2, SGNs, and SV

OHCs were counted under a 40X objective. Surviving OHCs were manually counted in regions of 8,11.3,16 and 22.6 kHz [72]. Select the same size regions containing 20 adjacent hair cells, then the OHCs survival percentage was calculated by subtracting out the number of the gaps (missing OHCs).

Immunolabeled signals of carboxyl-terminal binding protein 2 (CtBP2) on surface preparations were quantified from confocal images. Each image was captured with a 63X magnification lens under *z*-stack conditions with the same parameter settings, containing about 15 IHCs. CtBP2 immunofluorescence puncta per IHC was counted in regions of 5.6, 8, 11.3, 16, 22.6, and 32 kHz using Imaris software (Bitplane AG, Zurich, Switzerland).

Morphology of stria vascularis was assessed by quantifying H&E stained cross-sectional areas of each turn. SGC density in Rosenthal’s canal from apical to basal turn was computed by dividing the number of neurons by the cross-sectional area.

The cross-sectional area of Rosenthal’s canal was quantified by using CaseViewer software (3DHISTECH Ltd. Budapest, Hungary). Viable neurons with a clear round nucleus and homogeneous cytoplasm were included in the counts. Six independent sections per group were included in the statistics and were calculated for the SGN density (cells per square millimeters) of each turn separately.

### Whole-cell patch clamp recordings

Whole-cell patch clamp recordings were performed in IHCs at the apical-middle turn of the OC explant, estimated to be corresponding to frequencies of 8-16 kHz. Recording micropipettes were made from borosilicate glass tubes (1B150-4, World Precision Instruments, Inc., Sarasota, FL, USA) by using a horizontal pipette puller (Model P2000, Sutter Instruments, Novato, CA, USA). The recording pipettes were coated with M-Coat D (Vishay Measurements Group, CA, USA), with pipette resistances ranging from 4–6 MΩ. All recordings and analyses were conducted with jClamp software (http://www.SciSoft.com, New Haven, CT, USA), with an Axopatch 200B patch clamp amplifier and Digidata 1440B interface (Molecular Devices, LLC., San Jose, CA, USA). The extracellular solutions contained (in mM): 130 NaCl, 2.8 KCl, 10 CaCl_2_, 1 MgCl_2_, 10 HEPES. The pipette solution was 135 Cs-methanesulfonate, 10 CsCl, 10 TEA-Cl, 10 HEPES, 2 EGTA, 3 Mg-ATP and 0.5 Na-GTP. The osmolality was adjusted to 300 mOsm with D-glucose and pH 7.2~7.3 with NaOH. Liquid junction potential was corrected offline. Recordings were performed at room temperature (~23 °C).

A voltage ramp from −80 mV to +70 mV was applied to IHCs to evoke a Ca_2+_ current, and the peak of this Ca^2+^ current (I_Ca_) was determined. We then fitted the I-V relationship with a Boltzmann equation as below, allowing us to obtain the half activation potential (V_half_) and the slope of activation (*k*) which reflects the electrophysiological properties of calcium channels.

$$\text{I}(\text{V})=(\text{V}-{\text{V}}_{\text{rev}})\times \frac{{\text{G}}_{\text{max}}}{1+\exp(-(\text{V}-{\text{V}}_{\text{half}})\text{/}k)}$$

Whole-cell membrane capacitance measurements (C_m_) in IHCs were measured by using two-sine (70 mV peak to peak at both 390.6 and 781.2 Hz) superposed on the holding potential (-80 mV). The change of averaged C_m_ before and after stimulation (ΔC_m_ = C_m (response)_- C_m (baseline)_) was used to assess exocytosis of synaptic vesicles from IHCs, where series of depolarizing pulses with durations of 10 ms, 30 ms, 50 ms, and 100 ms were used. ΔC_m_ was measured and compared with Ca^2+^ current charge (Q) to evaluate vesicle release.

### Protein extraction and Western blotting

At least five mice (10 cochleae) for each genotype were sacrificed and pooled for Western blotting. Cochleae were rapidly dissected in ice-cold PBS. The tissues of sensory epithelia, stria vascularis and modiolus were gathered in a tube, with ice-cold RIPA lysis buffer plus protease inhibitor cocktail (78430, Thermo Scientific, Rockford, IL, USA) and phosphatase inhibitors. Samples were homogenized and centrifuged at 10,000 × g at 4°C for 20 minutes then the supernatants were collected. Protein concentration was measured by BCA method. 5 × SDS sample loading buffer was added to the samples, and then boiled for 10 minutes. Proteins were fractionated by 10%SDS-polyacrylamide gels electrophoresis for 60 minutes at 100V and blotted onto a Polyvinylidene Fluoride (PVDF) membrane (FFP33, Beyotime, Shanghai, CN) by electrical semi-dry transfer with 300mA current for 60 minutes. The membranes were rinsed with blocking buffer for 1 hour and then incubated overnight at 4°C in the desired primary antibodies. Antibodies listed below were purchased from Cell Signaling Technology, Inc. (Beverly, MA, USA): anti-Bcl2 rabbit mAb at 1:500 (3498), anti-Bax (2772) at 1:500, anti-Cytochrome C (4272) at 1:1000, cleaved caspase-3 Rabbit mAb (9664) at 1:750, anti-AMPKα (2532) at 1:1000, anti-p-AMPKα (2535) at 1:1000, anti-mTOR (2972) at 1:500, anti-p-mTOR (2971) at 1:500, anti-ULK1 (8054) at 1:500, anti-p-ULK1 (5869) at 1:500, anti-Sestrin2 rabbit mAb(8487) at 1:1000. The following primary antibodies were obtained from Abcam (Cambridge, England, UK): anti-AMPKα1 (ab32047) at 1:1000, anti-4hydroxynonenal (ab46545) at 1:500. Primary antibodies also included anti-BECN1 (NB500-249, Novus Biologicals, Inc., Littleton, Colorado, CO, USA) at 1:1000 and anti-GAPDH Mouse Monoclonal Antibody (AF0006, Beyotime, Shanghai, CN) at 1:5000 respectively, followed by the corresponding horseradish peroxidase-conjugated secondary antibodies at a dilution of 1:500. The protein bands were detected with the aid of enhanced chemiluminescence buffer using a luminescent image analyzer Amersham Imager 600 (GE Healthcare, Uppsala, Sweden). The relative densities of protein were calculated using ImageJ software (Wayne Rasband, National Institutes of Health, Bethesda, Maryland, USA).

### Noise exposure

Two-month-old healthy age-and-gender-matched C57BL/6J and AMPK^+/-^ mice littermates were selected in the study. Acoustic overexposure was conducted in a double-walled soundproof chamber. Unrestricted mice were placed in pairs in a wire cage on a heating pad (Harvard Apparatus, 55-7020). A free-field speaker was positioned 10cm away from the ears, with a broadband frequency noise ranging from 8-16 kHz for 2 hours at 106 dB SPL to induce permanent threshold shifts (PTS). ABRs were recorded at day0 (baseline), day1, day7, and day14 post noise exposure. ABR wave I of baseline and 14 days post noise were compared for amplitudes and latencies.

### Statistical analysis

Statistical differences were evaluated by one or two-way analysis of variance (ANOVA), followed by Bonferroni post-hoc comparison test for multiple genotype groups means that involved in frequency, sound intensity and regions of the cochlea, using GraphPad Prism 7.0 software (GraphPad, San Diego, US). Data were expressed as mean ± standard error of the mean (SEM) where values of p<0.05 indicate statistically significant. N represents the number of animals.
